# Die ökonomische Situation von Familien zwischen März und Mai 2020, den ersten zwei Monaten der COVID 19-Pandemie

**DOI:** 10.1007/s41025-021-00211-w

**Published:** 2021-02-23

**Authors:** Christina Boll

**Affiliations:** Abteilungsleitung Familie und Familienpolitik, Deutsches Jugendinstitut e. V. (DJI), 81541 München, Deutschland

**Keywords:** Wirtschaftliche Situation, Lockdown, Einkommen, Eltern, Arbeitsteilung

## Abstract

Der Artikel gibt einen Überblick über die wirtschaftliche Situation von Familien von Mitte März bis etwa Mitte Mai 2020, also den ersten zwei Monaten der COVID 19-Krise. Die Familieneinkommen sind wegen Arbeitslosigkeit und Kurzarbeit akut gefährdet. Auf der Einkommensseite greifen die Entschädigungen für Entgeltausfall durch Kita- und Schulschließungen im Rahmen des Infektionsschutzgesetzes nur partiell. Auch das gesetzliche Kurzarbeitergeld hilft nur bedingt, da Eltern unterschiedlich stark und manche gar nicht von ihm profitieren können, und die beschlossene Aufstockung kommt den Familien erst zeitverzögert zugute. Auch staatliche Familienleistungen wie der Notfall-Kinderzuschlag oder die jüngst beschlossene Elterngeldreform können die Einkommenseinbußen der Familien nur teilweise auffangen. Auf der Ausgabenseite drücken die Familien unveränderte Fixkosten. Zu den finanziellen Sorgen kommt in den Familien enorme Belastungen: Familien müssen Kinderbetreuung, Home Schooling, Haus- und Erwerbsarbeit allein schultern. Es zeichnet sich ab, dass Frauen weiterhin die Hauptlast der unbezahlten Arbeit tragen. Die jüngst vereinbarte Verlängerung der Entschädigungszahlungen nach dem Infektionsschutzgesetz wird den Familien helfen. Ein stufenweises Wiederhochfahren von Kitas, Schulen und Wirtschaft bleibt jedoch essenziell für die Rückkehr der Eltern in das Erwerbsleben und damit für die Sicherung der materiellen Existenzgrundlage von Millionen Familien.

## Abstract (en)

The article discusses German families’ economic situation in the first months of the current COVID 19 crisis from mid-March to mid-May, 2020. Family incomes are at high risk because of unemployment and short-time work. Recently introduced adjustments of the “Infektionsschutzgesetz” (Protection Against Infection Act) that established a claim for wage replacements for parents who have to provide childcare and home schooling for their children, due to the lockdown of kindergartens and schools, only partially compensate incurred losses. Further, far from all parents are entitled to the statutory short-time allowance, nor do recipients benefit to the same extent, and the approved upgrade of the replacement rate will only gradually become active. Moreover, public family-related measures such as the “Notfall-Kinderzuschlag” (emergency child supplement) and facilitations concerning parental leave entitlements during the crisis will only partly absorb the experienced income reductions. On the other hand, families’ daily expenses remain unchanged. While struggling how to make ends meet, families are confronted with a second burden, the challenge to reconcile domestic needs with job requirements. As first evidence suggests, women will bear the lion’s share of the crisis’ extra unpaid work for families. A prolongation of wage replacements for some extra weeks will relieve parents’ financial concerns, but a stepwise reopening of the child and youth welfare system, schools and the business sector remains essential for parents’ ability to return to work and to sustain their livelihood.

## Die Familieneinkommen sind wegen Arbeitslosigkeit und Kurzarbeit akut gefährdet

Die Familieneinkommen sind akut gefährdet, weil im Zuge der Krise vielen Eltern Arbeitslosigkeit oder Kurzarbeit droht. Nach Angabe der Bundesagentur für Arbeit (2020) führt die Corona-Krise zur schwersten Rezession in der Nachkriegsgeschichte. Das ifo-Institut (2020) prognostiziert einen Einbruch des Bruttoinlandsprodukts im zweiten Quartal 2020 um 12,2 %. Allein im März und bis 26. April gingen bei der Bundesagentur für Arbeit (BA) Anträge auf Kurzarbeit für bis zu 10,1 Mio. Personen ein, das ist das Dreifache der Anträge für das gesamte Krisenjahr 2009, so die BA. Die Arbeitslosenzahl ist im April gegenüber dem Vormonat saisonbereinigt um 373.000 und gegenüber dem Vorjahresmonat um 415.000 Personen gestiegen. Im Unterschied zur letzten Krise 2008/09 sind diesmal auch die Dienstleistungen, nicht nur die Industrie, stark vom Shutdown betroffen. Unter den Dienstleistungsbranchen, die die größten Geschäftseinbrüche melden (Tourismus, Gastgewerbe, Kultur, Gesundheitswesen und Luftfahrt), sind viele frauendominierte Branchen. Hinzu kommt, dass auch der Sektor Erziehung und Unterricht einen Frauenanteil von 71 % aufweist. Männerdomänen sind hingegen das Verarbeitende Gewerbe, Bau, Verkehr und Lagerei. Zugleich stellen Frauen einen Anteil von 77 % im Gesundheits- und Sozialwesen, in dem viele systemrelevante Berufe ausgeübt werden (Bundesagentur für Arbeit 2019, S. 12).

## Entschädigungen für Entgeltausfall durch Kita- und Schulschließungen greifen nur partiell

Mit Beginn des Lockdowns von Schulen und Kindergärten hat die Große Koalition noch im März durch eine Erweiterung des Infektionsschutzgesetzes (§ 56 IfSG) eine Entschädigungsleistung für Sorgeberechtigte aufgenommen, die den Verdienstausfall von Eltern mit Kindern unter 12 Jahren teilweise ausgleichen soll (Deutscher Bundestag 2020a). Allerdings wird nur entschädigt, wer keine andere Betreuung findet, wer nicht im Homeoffice arbeiten kann und wer keine anderweitige Entschädigung, z. B. durch das gesetzliche Kurzarbeitergeld, erhält. Zudem werden nur 67 % des Nettoeinkommensausfalls ersetzt; dies ist ein Problem für Familien mit kleinen Einkommen, die auch schon in Normalzeiten jeden Cent zweimal umdrehen müssen. Die Verlängerung des Entschädigungsanspruches für Eltern nach § 56 Infektionsschutzgesetz (IfSG), die das Bundeskabinett am 20.04.2020 beschlossen hat (BMAS 2020a), ist konsequent angesichts der Tatsache, dass Kitas und Schulen nur sehr langsam und stufenweise wieder geöffnet werden können. Einen Antrag der FDP-Fraktion (Deutscher Bundestag 2020b), der auch eine Verlängerung der sechswöchigen Bezugsdauer der Entschädigungszahlungen nach dem Infektionsschutzgesetz sowie eine Corona-Elternzeit und eine Kitagebührenbefreiung von Eltern für die Dauer der Pandemie beinhaltete, hatte der Deutsche Bundestag am 06.05.2020 abgelehnt.

## Das Kurzarbeitergeld greift ebenfalls nur teilweise und zeitverzögert; zudem dürften Frauen weniger als Männer profitieren

Im internationalen Vergleich hat Deutschland in der letzten Krise 2008/09 vor allem durch das Instrument der Kurzarbeit viele Jobs retten können, die ansonsten aufgrund des immensen Nachfrageausfalls verloren gegangen wären (Münstermann 2012). Gesetzliches Kurzarbeitergeld steht aber nur sozialversicherungspflichtig Beschäftigten zu. Selbstständige und Minijobber*innen sind nicht anspruchsberechtigt.

Im gewerblichen Bereich sind etwa 70 % der rentenversicherungspflichtigen Minijobber Frauen. Im Privathaushalt beläuft sich dieser Anteil auf etwa 90 % (Deutsche Rentenversicherung Knappschaft Bahn-See/Minijobzentrale 2019, S. 2). Besonders gravierend ist die wirtschaftliche Lage für die 4,7 Mio. ausschließlich geringfügig Beschäftigten ohne aktuelle Beschäftigung; von diesen sind 62 % Frauen (Bundesagentur für Arbeit 2019, S. 7). Gut ist, dass mit der Kurzarbeitergeldverordnung vom 25.03.2020 (BMAS 2020b) beschlossen wurde, dass das Kurzarbeitergeld nun auch für Beschäftigte in Leiharbeit beantragt werden kann und dass die Mitarbeiterzahl, die von einem Entgeltausfall betroffen ist, auf 10 % abgesenkt wurde. Das verbreitert den Kreis der Anspruchsberechtigten und hilft auch den Familien. Für diejenigen Eltern, die Anspruch auf Kurzarbeitergeld haben, gleicht es allerdings nur 67 % des Nettoentgeltverlusts aus, der durch die Arbeitszeitreduktion zustande gekommen ist. Dies ist im europäischen Vergleich ein eher geringer Anteil (Schulten und Müller 2020, S. 8). Wiederum trifft der Entgeltverlust Familien, die auch bei regulären Arbeitszeiten der Eltern nur ein kleines Einkommen haben, besonders hart.

Frauen verdienen insgesamt im Durchschnitt 20 % weniger als Männer (Statistisches Bundesamt 2020a). Sie sind auch eher von Niedrigeinkommen betroffen: Wie eine Auswertung der BA-Beschäftigtenstatistik zeigt, erzielten 24,9 % (13,9 %) der in sozialversicherungspflichtigen Vollzeitjobs beschäftigten Frauen (Männer) im Jahr 2016 ein monatliches Bruttoeinkommen von bis zu 2000 € (Hobler et al. 2020, S. 23). Daher werden Frauen auch vom Kurzarbeitergeld betragsmäßig weniger profitieren als Männer. Hinzu kommt, dass Frauen seltener in den Genuss einer tarifvertraglich geregelten betrieblichen Aufstockung des Kurzarbeitergeldes kommen (WSI 2020). Denn Frauen arbeiten häufiger als Männer in kleinen Betrieben, die seltener von der Tarifbindung erfasst sind. Dies hat Folgen: Während nur 13 % der Eltern mit Kindern unter 15 Jahren, die in Betrieben mit mindestens 300 Beschäftigten arbeiten, einen deutlichen Rückgang des Haushaltseinkommens hinnehmen müssen, gilt dies für 17 % der Beschäftigten in Kleinstbetrieben unter 10 Beschäftigten. Dies ergab eine repräsentative Online-Befragung des Instituts für Demoskopie Allensbach im Auftrag des BMFSFJ unter 1493 Eltern mit Kindern im Zeitraum 16.04. bis 03.05.2020 (BMFSFJ 2020a). Das durchschnittlich niedrigere Einkommen von Frauen im Vergleich zu Männern, das die Folge eines geringeren mittleren Stundenlohns und einer niedrigeren mittleren Wochenstundenzahl ist, ist insbesondere für alleinerziehende Mütter problematisch, da hier kein zweites Einkommen im Haushalt kompensieren kann.

Zwar hat die Große Koalition am 22.04.20 im Rahmen des Sozialschutzpakets II nicht nur eine einmalige Verlängerung der Anspruchsdauer des Arbeitslosengeldes nach SGB III für diejenigen, deren Anspruch zwischen dem 1. Mai 2020 und dem 31. Dezember 2020 enden würde, beschlossen, sondern sich auch auf eine zeitlich gestaffelte Anhebung des Kurzarbeitergeldes geeinigt: Ab Monat 4 bzw. 7 des Bezugs von Kurzarbeitergeld soll es für Familien auf 77 bzw. 87 % (und auf 70 bzw. 80 % für alle anderen) angehoben werden, befristet bis zum 31.12.2020 (BMAS 2020c). Für die ersten drei Monate müssen die Familien also mit 67 % Lohnersatz auskommen, oder aber sie müssen Leistungen der sozialen Mindestsicherung beantragen. Allerdings geben in einer Beschäftigtenbefragung 40 % der Personen, die derzeit schon in Kurzarbeit sind, an, diese Situation finanziell maximal drei Monate durchstehen zu können (Hans-Böckler-Stiftung 2020). Eine repräsentative Studie des Meinungsforschungsinstituts Kantar hat, wie die WELT AM SONNTAG (2020) berichtet, ergeben, dass ein Drittel (30,4 %) der Haushalte mit drei Personen mit weniger Geld als vor der Krise auskommen müssen, hingegen nur 17,1 % der Single-Haushalte. Allerdings sind Alleinlebende von Alleinerziehenden deutlich zu unterscheiden: Alleinerziehende mit Kindern unter 15 Jahren im Haushalt waren von Einkommensverlusten stärker betroffen als Paarfamilien mit gleichaltrigen Kindern. Mit 24 % gab ein höherer Anteil der Alleinerziehenden als von Eltern in Paarbeziehungen (18 %) ein deutlich gesunkenes Haushaltseinkommen an (BMFSFJ 2020a).

Zwar hat die Bundesregierung auch die Möglichkeiten des Zuverdiensts für alle Beschäftigten in Kurzarbeit ab dem 1. Mai bis zum 31. Dezember 2020 für alle Berufe gelockert: Es darf in diesem Zeitraum bis zur vollen Höhe des bisherigen Monatseinkommens hinzuverdient werden (BMAS 2020c). Für Eltern in Elternzeit werden aufgrund der Pandemie erhaltene Entgeltersatzleistungen nicht auf das Elterngeld angerechnet (BMFSFJ 2020b). Jedoch dürfte die Chance zum Nebenerwerb aufgrund der zeitlichen Mehrfachbelastungen der Eltern in der Krise mit Betreuung und Home Schooling eher von wenigen Eltern und insbesondere wenigen Müttern genutzt werden können.

## Zugleich ändert sich nichts an den Fixkosten: Die Mieten müssen bezahlt werden, steigende Lebensmittepreise erschweren die Situation zusätzlich

Trotz geringerer Einkommen müssen Familien unverändert die Fixkosten wie beispielsweise Miete, Strom und Versicherungen weiterhin tragen. Als wäre dies nicht schon genug, leiden Familien, insbesondere jene mit nur bescheidenen Einkommen und kinderreiche Familien, ganz besonders unter steigenden Lebensmittelpreisen, die die Kaufkraft des ohnehin geschrumpften Einkommens weiter schmälern. Die Verbraucherpreise für Nahrungsmittel sind im April 2020 gegenüber dem Vorjahresmonat um 4,8 % gestiegen, im Januar waren es noch 2,3 % (Statistisches Bundesamt 2020b). Dass dieser Preisauftrieb bei Lebensmitteln nicht allein Corona-bedingt ist, hilft den Familien indes wenig. Immerhin beinhaltet das Sozialschutzpaket II auch die Regelung, dass Schul- und Kitakinder im Rahmen der Leistungen des Bildungspakets weiterhin mit Mittagessen versorgt werden können.

## Zu den finanziellen Sorgen kommt in den Familien eine enorme zeitliche und psychische Belastung

Die zunehmend angespannte finanzielle Situation trifft die Familien zusammen mit den enormen Herausforderungen des Zeitmanagements. Der schon in Normalzeiten herausfordernde Spagat vieler Mütter zwischen bezahltem Job und der traditionell bei ihnen angesiedelten Hauptlast der Hausarbeit und Kinderbetreuung wächst jetzt im Krisenmodus auf eine Vierfachbelastung aus Erwerbsarbeit, Hausarbeit, Kinderbetreuung 24/7 und Home Schooling an. Dies übersteigt das Machbare. Viele Mütter, aber auch Väter, sind physisch und psychisch an ihrer Belastungsgrenze oder bereits jenseits derselben. Das gilt insbesondere für Alleinerziehende, die alle Lasten allein schultern müssen. On top’ kommen für Trennungsfamilien Aushandlungsprozesse mit dem Ex-Partner bzw. der Ex-Partnerin zur Betreuung der gemeinsamen Kinder, die in den sich dynamisch veränderten Arbeitsanforderungen von Eltern permanent neu ausjustiert werden müssen. Da Großeltern wegen des Kontaktverbots als Betreuungspersonen ausfallen, sind Eltern hier auf sich allein gestellt. Zwar sind viele Jobs Homeoffice-fähig und Corona bringt hoffentlich nachhaltige Lerneffekte zur Produktivität in den eigenen vier Wänden, in denen Menschen, darunter viele Eltern, derzeit – zusammen mit den Menschen in systemrelevanten „outdoor“-Berufen – die Wirtschaft und Verwaltung der Republik am Laufen halten. Dies geht jedoch nur durch eine massiv erhöhte zeitliche Flexibilität, denn Care-Leistungen für Kinder und pflegebedürftige Angehörige lassen sich nicht in den Feierabend verschieben. Die Wirkungen des Homeoffice auf die Arbeitsteilung zur Kinderbetreuung sind dabei komplex: Arbeitet der Vater im Homeoffice, reduziert sich die Wahrscheinlichkeit der mütterlichen Alleinbetreuung, sie steigt jedoch, wenn die Mutter im Homeoffice arbeitet. Diese Zusammenhänge gelten auch für hochgebildete Eltern (Zoch et al. 2020). Die hohen Opportunitätskosten hochgebildeter Frauen sind es also nicht allein, die die Arbeitsteilung prägen; vielmehr müssen die Markt- und Haushaltsproduktivitäten der Partner in Relation zueinander, die Ausgangskonstellation des Paares vor der Krise und die Geschlechternormen berücksichtigt werden (Boll und Schüller 2020).

Bei drei Vierteln der erwerbstätigen Eltern ist die Belastung durch Kinderbetreuung in der Covid-19-Pandemie gestiegen, wie 2889 Eltern einer Online-Befragung von sozialversicherungspflichtig beschäftigen Eltern mit Kindern bis 18 Jahren im Haushalt zwischen dem 8. und 25. Mai 2020 ergab; die Belastung stieg dabei bei Akademikern stärker als bei anderen Berufsgruppen und hier bei den Müttern noch stärker als bei den Vätern (Fuchs-Schündeln und Stephan 2020). Die Belastung der Eltern ergibt sich nicht nur in zeitlicher Hinsicht, sondern auch durch die Sorgen, wie ihre Kinder die Krise bewältigen können. Auch in den Bewältigungsstrategien von Kindern zeigen sich jedoch soziale Disparitäten: Leistungsstärkere Kinder sind deutlich motivierter, lernen zuhause aber auch unter günstigeren Bedingungen als leistungsschwächere, deren Mutter zudem seltener einen akademischen Abschluss hat (Huebener und Schmitz 2020). Schulkinder aus Elternhäusern mit höheren Einkommen sind wiederum engagierter beim eLearning (Chetty et al. 2020). Kinder aus sozial bessergestellten Elternhäusern haben offenbar bessere Ausgangsbedingungen, mit der Krise zurecht zu kommen. Dazu passt, dass Eltern mit einer angespannten finanziellen Situation die Krisenbelastung für ihre Kinder deutlich höher einschätzen als diejenigen, die ihre finanzielle Lage positiver beurteilen (Langmeyer et al. 2020).

## Es ist offen, ob Corona für die Arbeitsteilung im Paar Fortschritte oder Rückschritte bringen wird (vgl. Boll 2020)

Ob Eltern die bezahlte und unbezahlte Arbeit nach Corona gleicher oder noch ungleicher aufteilen als zuvor, wird von mehreren Faktoren abhängen. Ein Hebel könnte die monetäre Aufwertung von Frauenberufen sein, die in der Krise erhöhte Aufmerksamkeit erfahren, weil darunter viele sogenannte systemrelevante Berufe sind. Wenn sich die beklatschten Leistungen in Gesundheits‑, Pflege- und Sozialberufen nach der Krise in höhere Stundenlöhne umsetzen, senkt das den Gender Pay Gap. Dies würde nicht nur die Familieneinkommen erhöhen, sondern über die gesteigerten Erträge der Müttererwerbstätigkeit auch einen Anpassungsdruck auf die innerhäusliche Arbeitsteilung entfalten^1^. Hier ist der Staat als Arbeitgeber gefragt, mit gutem Beispiel voranzugehen und die Tarifierungs- und Arbeitsbewertungsverfahren im öffentlichen Dienst auf Geschlechterneutralität zu überprüfen (Boll und Lagemann 2018). In eine ähnliche Richtung könnte der Digitalisierungsschub gehen, den Corona zweifelsohne bringen wird: Eine höhere Legitimation und Verbreitung von zeit- und ortsflexiblem Arbeiten und ein weiteres Zurückdrängen reiner Präsenzkulturen wird insbesondere den beruflichen Karrieren von Frauen und Müttern helfen, was wiederum tendenziell die Geschlechterlohnlücke senkt. Frauen haben ein noch höheres unausgeschöpftes Homeoffice-Potenzial als Männer (Alipour et al. 2020), das nun gehoben werden könnte. Jenseits dieser eher auf der betrieblichen Ebene erwarteten Lerneffekte könnte der Geschlechtergleichstellung auch ein Lerneffekt im häuslichen Bereich helfen, wenn nämlich Väter in Homeoffice-fähigen Berufen jetzt zuhause verstärkt „mit anpacken“ und dadurch neue Verhaltensmuster eingeübt werden. Insbesondere dann, wenn Mütter in systemrelevanten Berufen aus dem Haus müssen, könnte Corona hier einen Rollentausch erzwingen. Ob diese Lerneffekte nachhaltig sind, wird u. a. von der Dauer der Krise abhängen. 67 % der Deutschen im Alter 18 bis 69 rechnen mit sehr positiven (23 %) oder eher positiven (44 %) Auswirkungen von Corona auf die Organisation der Berufsarbeit, z. B. Homeoffice (Sinus Institut 2020). Zudem kommt es für eine Veränderung der Arbeitsteilung nicht nur auf das zusätzliche Zeitbudget der Väter, sondern auch auf jenes der Mütter an sowie darauf, wer im Paar das meiste Geld nach Hause bringt (Boll und Schüller 2020). Erste Befunde deuten eher darauf hin, dass Frauen in der laufenden Krise mehr statt weniger der unbezahlten Arbeit schultern (Langmeyer et al. 2020). Berufstätige Mütter übernehmen die Betreuung ihrer Kinder häufig allein, während viele Väter ihre Kinder nur ergänzend betreuen (Zoch et al. 2020). Dies könnte daran liegen, dass Eltern in der Krise wenig „Nerven für Experimente“ haben und eher auf bewährte Routinen zurückgreifen – und dies bedeutet in weiten Teilen der Bevölkerung quer durch alle gesellschaftlichen Schichten die Rückkehr zur traditionellen Arbeitsteilung. Die Zeit wird zeigen, welche Mechanismen sich letztlich durchsetzen, sprich: wer was aus der Krise lernt.

## Bilanz im Mai: Stufenweises Wiederhochfahren von Kitas, Schulen und Wirtschaft ist essenziell für die Sicherung der materiellen Existenzgrundlage von Millionen Familien

Zu Recht weisen Wohlfahrtsverbände, Wissenschaftler*innen und Praktiker*innen, denen das Wohl von Eltern und Kindern am Herzen liegt, auf die Gefahren eines langen Lockdowns von frühkindlicher Betreuung und Bildung für die kindliche Entwicklung, die psychische und physische Gesundheit von Kindern und Eltern und für die finanzielle Stabilität von Familien hin (u. a. AGF 2020; Liga Kind 2020). Daher brauchen Kinder und Eltern eine stufenweise Öffnung der *Kitas und Schulen *unter Berücksichtigung der epidemiologischen Lage. Vorschläge, wie solche Stufenpläne im Detail aussehen könnten, liegen auf dem Tisch (siehe bspw. den Aufruf namhafter Bildungsökonomen an die Politik vom 03.05.20, Danzer et al. 2020). Es ist nun an den Bundesländern, hier in die Umsetzung zu kommen, denn die Bundesregierung hat die Handlungskompetenz in diesem Bereich am 6. Mai 2020 an die Bundesländer delegiert. Neueste Berechnungen des Forschungsverbund DJI/TU Dortmund zeigen, dass ohne Rückgriff auf Kita-Personal, das zur Risikogruppe gehört, etwa die Hälfte der Kinder zumindest mit einem reduzierten Stundenumfang in Kleingruppen in die Kitas zurückkehren könnten. Immerhin könnten hierbei jene Gruppen von Kindern, die besonders dringende Bedarfe haben, zu einem Gutteil berücksichtigt werden. Es wird jetzt von der Kreativität und dem Engagement der handelnden Akteure in Ländern und Kommunen abhängen, was möglich wird. Gut ist auch, dass laut Beschluss des Deutschen Bundestages vom 07.05.20 Elterngeldmonate aufgeschoben werden können, wenn Eltern in systemrelevanten Berufen und Branchen akut keine Elternzeit möglich ist. Zudem soll etwaige Mehrarbeit, die in der Krise in diesen Jobs nötig ist, den Partnerschaftsbonus nicht gefährden (BMFSFJ 2020c).

Wie lange Eltern noch Einkommenseinbußen drohen, hängt neben der Wiederöffnung von Kitas und Schulen auch davon ab, wie schnell die *Wirtschaft* wieder hochfährt, mithin Eltern ins Erwerbsleben bzw. aus der Kurzarbeit wieder auf ihren ursprünglichen Arbeitszeitumfang zurückkehren können. Nach dem Beschluss der Bundeskanzlerin und der Regierungschefinnen und Regierungschefs der Länder vom 6. Mai 2020 (Bundesregierung 2020) dürfen die Geschäfte sukzessive und unter Einhaltung der Auflagen wieder öffnen. Die schrittweise Öffnung von Gastgewerbe und dem Kulturbereich liegt seitdem im Entscheidungsbereich der Bundesländer. Dies wird aufgrund des regional unterschiedlichen Infektionsgeschehens in Deutschland unterschiedlich schnell der Fall sein. Auch haben die Branchen unterschiedliche Möglichkeiten, Infektionsschutzmaßnahmen zu ergreifen. Dies liegt nicht nur daran, dass in der industriellen Produktion Abstandsregeln leichter einzuhalten sind als beispielsweise bei personenbezogenen Dienstleistungen, sondern ist u. a. auch den unterschiedlichen personellen und finanziellen Ressourcen der Betriebe geschuldet. Gerade Klein- und Kleinstunternehmen tun sich hier teilweise schwer. Zugleich greifen auch staatliche Leistungen nur bedingt: Der Notfall-KIZ – der erleichterte Zugang zum Kinderzuschlag, den die Bundesregierung zur Abfederung Corona-bedingter finanzieller Einbußen von Familien mit kleinen Einkommen geschaffen hat – ist auf maximal 185 € pro Monat pro Kind begrenzt und kann die Einkommenseinbußen in vielen Familien nicht vollständig auffangen. Zudem schützt die am 07.05.20 beschlossene Elterngeldreform (BMFSFJ 2020c) zwar gegen den Verfall von Ansprüchen und eine Minderung des Elterngelds durch andere Entgeltersatzleistungen, die während der Pandemie an Eltern gezahlt werden; wegen des nur partiellen Lohnersatzes und weil nicht alle Eltern während des Elterngeldbezugs arbeiten, dürfte das Nettoeinkommensplus hieraus für die Gesamtheit der Familien dennoch beschränkt sein. Die am 14.05.2020 vom Bundestag beschlossene zeitliche Staffelung der Kurzarbeitergeldaufstockung (Sozialpaket II) greift erst ab Monat 4. Es ist daher zu begrüßen, dass der Staat den Familien mit der nun beschlossenen verlängerten Anspruchsdauer für die Entschädigungsregelung nach § 56 IfSG auf bis zu 10 Wochen, für Alleinerziehende bis zu 20 Wochen, unter die Arme greift und ihnen hilft, die Übergangsphase zu einer „neuen Normalität“ nach Corona zu überbrücken. Denn die Wirtschaft wird nur allmählich wieder in Schwung kommen, Kitas und Schulen können nur zeitverzögert wieder öffnen und Betreuung und Bildung wird auch in den kommenden Wochen eher sporadisch als verlässlich und regelmäßig erfolgen. Weiterhin bleibt eine vollumfängliche Rückkehr von Eltern in die Erwerbstätigkeit die Voraussetzung für die materielle Existenzsicherung von Familien.

## Footnotes

1 Hier wird mit einer Lohnveränderung innerhalb der Person im Längsschnitt argumentiert. Empirische Befunde auf Basis von Querschnittdaten für Mütter mit unterschiedlichen Lohnhöhen können zu anderen Ergebnissen kommen, v. a. auf Grund unbeobachteter Heterogenität.
